# Web-Based Targeted Nutrition Counselling and Social Support for Patients at Increased Cardiovascular Risk in General Practice: Randomized Controlled Trial

**DOI:** 10.2196/jmir.6.4.e44

**Published:** 2004-12-16

**Authors:** Marieke Verheijden, J Carel Bakx, Reinier Akkermans, Henk van den Hoogen, N Marshall Godwin, Walter Rosser, Wija van Staveren, Chris van Weel

**Affiliations:** ^3^Centre for Studies in Primary CareQueen's UniversityKingston ONCanada; ^2^Department of Family MedicineUniversity Medical Centre St RadboudNijmegenThe Netherlands; ^1^Division of Human NutritionWageningen UniversityWageningenThe Netherlands

**Keywords:** Internet, diet, nutrition counseling, social support, cardiovascular risk

## Abstract

**Background:**

Using the Internet may prove useful in providing nutrition counselling and social support for patients with chronic diseases.

**Objective:**

We evaluated the impact of Web-based nutrition counselling and social support on social support measures, anthropometry, blood pressure, and serum cholesterol in patients at increased cardiovascular risk.

**Methods:**

We conducted a randomized controlled trial among patients with increased cardiovascular risk in Canadian family practices. During 8 months, patients in the intervention group and control groups received usual care. Patients in the intervention group also had access to a Web-based nutrition counselling and social support tool (Heartweb). Site use during the study was monitored. We measured social support, body mass index, waist/hip ratio, blood pressure, and cholesterol levels at baseline and at 4 and 8 months to assess the effectiveness of the intervention.

**Results:**

We randomized 146 patients into the Web-based intervention (n=73) or the control group (n=73). Within the Web-based intervention group, Heartweb was used by only 33% (24/73) of patients, with users being significantly younger than nonusers (P=.03). There were no statistically significant differences between the intervention group and the control group in changes in social support, anthropometry, blood pressure, and serum cholesterol levels.

**Conclusions:**

Uptake of the Web-based intervention was low. This study showed no favourable effects of a Web-based nutrition counselling and social support intervention on social support, anthropometry, blood pressure, and serum cholesterol. Improvements in reach and frequency of site use are needed to increase the effectiveness of Web-based interventions.

## Introduction

General practitioners are in an ideal position to provide nutrition counselling to patients at increased cardiovascular risk. Unfortunately, they perceive barriers that limit their nutrition counselling practices such as lack of time, lack of skills, and lack of patient motivation [1]. Using the Internet may prove useful in overcoming barriers related to lack of skills and lack of time [2]. The Transtheoretical Model may provide a framework in which general practitioners can deal with different levels of patient motivation and has been used repeatedly to give shape to nutrition counselling in general practice [3-5]. The model distinguishes 5 different stages of readiness to change behaviour: precontemplation, contemplation, preparation, action, and maintenance. Further, the model includes processes of change, decisional balance, and self-efficacy [6]. Nutrition counselling efforts thus far have not shown much success in the long term. Social support, which plays a role in many of the processes of change in the Transtheoretical Model, may be particularly crucial in this respect [7-9]. Unfortunately, social support is often not included in nutrition counselling interventions. The limited research thus far has suggested that Internet applications may increase social support levels by providing a significant avenue by which peer interaction can take place [10-13], eg, by means of online patient groups, listservs, bulletin boards, and chat rooms. Internet applications obviously have other advantages: they require a 1-time-only effort for design and implementation, after which they may be useful for large groups of people.

The current study assessed the effectiveness of a Web-based nutrition counselling and social support program as an addition to usual care in Canadian patients at increased cardiovascular risk in general practice. The Transtheoretical Model was used as the theoretical framework for the intervention. Because this study addresses the effectiveness rather than the efficacy of our intervention, care was taken to ensure a study protocol that would be feasible in general practice. As a result, patients were not overly encouraged to use the study website. Outcome measurements were anthropometry, blood pressure, and serum cholesterol levels. We hypothesized that a Web-based nutrition counselling and social support program would lead to improved clinical outcomes in patients who used the program.

## Methods

### Study Participants

**Figure 1:**
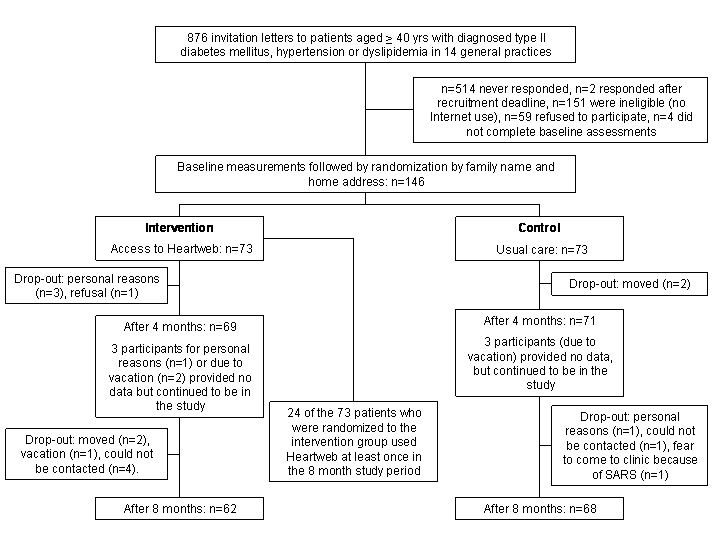
Selection and flow of participants

Fourteen general practitioners (including Dr Godwin, who is an author of this paper) with a total of 7944 patients in the Queen's University General Practice Centre in Kingston (Canada) agreed to bring the study to the attention of their patients who were at increased cardiovascular risk. The general practitioners sent letters to all 876 patients 40 years and older who appeared in the computerized billing system in the year before recruitment for at least 1 of the following: hypertension, type 2 diabetes mellitus, and dyslipidemia. To be eligible for the study, patients had to confirm the diagnosis of 1 or more of the aforementioned risk factors for cardiovascular disease and to report to use the Internet. We included 146 patients in the intervention study. Figure 1 provides details on the recruitment and flow of the participants.

### Procedure

At baseline, patients were contacted to schedule 2 appointments in their general practitioner's office. At the first appointment, patients' height and weight, blood pressure, and waist and hip circumferences were measured as described below. Patients were then referred to the phlebotomy laboratory of the adjacent hospital to have a blood sample taken. At the first appointment, patients were also given a study questionnaire. They were asked to return the questionnaire at the second appointment 1 week later. At the second appointment, patients only had to go the phlebotomy laboratory to have a blood sample taken. Follow-up assessments were scheduled 4 and 8 months after baseline. After completion of baseline assessments (September 2002 to December 2002), an independent researcher randomly assigned all 146 patients to the intervention group or the control group by using a computerized table. The control and intervention groups each comprised 6 pairs of participants (12 individuals) living at the same address and/or with the same surname. Patients within each pair were randomized into the same group to avoid contamination of the information within families or households. The randomization procedure resulted in 73 patients in the intervention group and 73 patients in the control group. Patients in the control group received usual care. In addition to usual care, patients in the intervention group were given a personal registration code for the password-protected access to a Web-based nutrition counselling and social support program (Heartweb) that was specifically designed for this study. After 4 months, patients were sent a reminder of their registration code. At each time point, patients in the intervention group and those in the control group were sent result sheets with their body mass index, blood pressure and cholesterol values. As requested by the Queen's University Human Research Ethics Board, result sheets were also sent to patients' respective general practitioners.

Patient blinding in nutrition counselling trials is difficult to achieve because patients in the intervention group may well be aware of the extra attention they receive. From our previous intervention study [5], however, it appeared that patients in both groups thought they had been in the intervention arm of the study because of all the physical assessments and the questionnaires. Most current treatment guidelines for patients at increased cardiovascular risk suggest nutrition and other lifestyle counselling, which should have made nutrition counselling a familiar notion for patients in both groups. Similar to the previous study, patients in the current study were told that the study was aimed at assessing nutrition-counselling practices in general practice. We anticipated that, as a result, patients in the control group and those in the intervention group would think they had been randomized into the intervention group.

The Human Research Ethics Board of Queen's University approved the study protocol and written informed consent was obtained from all participants. To assess the external validity of our trial, a random sample of 146 patient records was drawn from the nonresponders. These records were reviewed for blood pressure and lipid profile data from assessments between August 1, 2001 and August 1, 2002. Data were compared with baseline values of study participants.

### The Intervention

As was previously done in other (Web-based) intervention studies [5,14-16], Heartweb included a procedure to target counselling messages to patients' readiness to decrease their fat consumption. Dietary fat reduction was chosen because of its key role in decreasing cardiovascular risk. We defined readiness to decrease fat consumption on an operational level using the Stages of Change Model [6].

**Figure 2:**
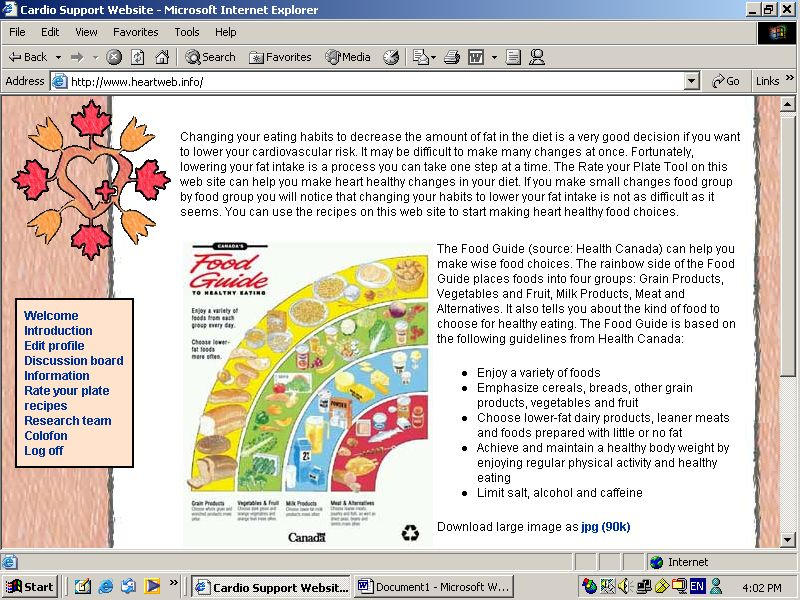
Stage-matched nutrition information for the preparation stage

Once every month, Heartweb presented patients with a short assessment tool to determine their stage of change. Patients were then automatically presented with an information package for that particular stage of change. Figure 2 shows an example of the personalized targeted counselling program. No longitudinal component was built into the site, ie, when patients logged on to the site a second time, no reference was made to their first “results.” If patients did not progress through the stages, they were presented with the same information package twice. Once they were presented with the information package for 1 stage of change, patients could browse to the packages for the other stages of change. The targeted information packages were designed to create or enforce a positive attitude towards decreasing fat consumption, to make people aware of the risks associated with increased fat intakes, and to provide patients with practical advice on how to decrease fat consumption. Canada's Food Guide to Healthy Eating (Minister of Supply and Services Canada 1992; catalog number H39-252/1992E) and existing Web-based and non-Web-based materials were used during the development of the Web-based targeted intervention packages that constituted Heartweb [2-5,17-30]. In the precontemplation stage, for example, people were made aware of their problem behaviour and of the possible link between diet and the diagnosis of hypertension, type 2 diabetes mellitus, and dyslipidemia. They were also informed about the possibility to change and were encouraged to consider implementing dietary changes. Care was taken to avoid being patronizing. Common misconceptions about one's dietary behaviour could result in people classifying themselves in action or maintenance while not eating a sufficiently low-fat diet. This has consequences for the personalized feedback messages. In the action stage, people are usually encouraged to continue their efforts towards behaviour change (ie, further changes are often recommended). In the maintenance stage, by contrast, people are usually encouraged to maintain their current diet and no further changes are recommended. To limit the possibility of inappropriately doing so, people in the maintenance stage were presented with a short checklist designed to assess whether or not patients were likely to be truly eating a sufficiently low-fat diet [31]. Patients who were truly eating a low-fat diet were given the appropriate reinforcement. Patients who were most likely not eating a low-fat diet were given feedback on this possible misconception and were asked to reconsider dietary changes. This provided a means to ensure appropriate feedback even for those who mistakenly thought they were eating a low-fat diet.

Independent of peoples' stage of change, we included a self-assessment tool for dietary fat intake provided by the Nutrition Promotion Program of the Kingston, Frontenac and Lennox & Addington Health Unit to increase patients' awareness of their dietary behaviour [32]. To increase peoples' confidence in their ability to adopt a low-fat diet, we added 4 heart-healthy recipes provided by the Dietitians of Canada to the web site. For more healthy recipes, patients were referred to web sites of the Canadian Heart and Stroke Foundation and the Dietitians of Canada. Heartweb also consisted of a bulletin board that enabled patients to post messages for social support. To encourage the use of the bulletin board, the information packages for each of the stages of change concluded by referring participants to the bulletin board. Using a patient entry, we posted messages on the discussion board to get the online conversation started and to keep it going when it slowed down. The research team participated in the online discussion only when specifically requested by the participants.

Data on date, time, and duration of site use and the answers to the online Stages of Change questionnaire and the self-assessment tool for dietary fat intake were stored on the web site and were accessible only to the research team.

### Outcome Measurements

Outcome measurements were assessed at baseline and 4 and 8 months after randomization by using the procedures described below. Measurements were conducted by researchers who were blinded to the outcomes of the randomization procedure.

Participants were asked to fill in a questionnaire consisting of a general section that included items on demographic data, smoking status, physical activity, Internet use, and medications. Stage of readiness to decrease fat intake was assessed with an algorithm [5]. The social support section consisted of a version of the 16-item social support scale used by Winzelberg et al [11] to measure perceived social support that was adapted to be applicable for a heart-healthy diet. The questionnaire included specific items on the exchange of online support. The availability and use of a social support network was measured with the 7-item National Population Health Survey social support scale [33]. The introduction to this scale specifically requested patients to report personal, phone, mail, and email contact. Participants were also asked to complete a food frequency questionnaire [34] to assess nutrient intakes. In contrast to previous publications [34], we found high (partial) noncompletion rates. Patients were contacted by phone and/or by mail to obtain complete data. Despite this contact, reporting of unrealistically low total energy intakes was frequent (<1000 kcal = 4200 kJ). Therefore dietary intake data are not discussed in the current paper.

We measured body weight (to the nearest kilogram) by using a Healthometer (model 134KGS HOM 2599). Similar scales were in use in all clinics throughout the University General Practice Centre. Body height, waist circumference, and hip circumference (to the nearest centimetre) were also measured at the practice center. All measurements were conducted without shoes or heavy clothing. Body mass index (BMI, kg/m^2^) and waist-to-hip circumference ratio were calculated. Blood pressure was measured in the sitting position 4 times on each occasion by using an auto inflate blood pressure monitor (UA-767, A&D Engineering). Readings were conducted on the same arm for baseline and follow-up measurements. The mean of the last 3 readings was used for analyses.

We measured fasting serum levels of total cholesterol, high-density lipoprotein cholesterol (HDL), and triglyceride in 2 blood samples taken with a 1-week interval. The mean of the 2 samples was used for analyses. All analyses were conducted at the laboratory of the Kingston General Hospital (Kingston, Canada) by using the Roche Modular System (Roche Diagnostics, a division of Hoffmann-La Roche). Low-density lipoprotein cholesterol (LDL) was calculated according to the formula of Friedewald et al [35]. When triglyceride levels exceeded 4.52 mmol/L, no LDL levels were calculated.

After completion of the study, patients were asked to fill in an evaluation questionnaire. For patients in both groups, this questionnaire contained items on the organization of the study and contact with the research team. For patients in the intervention group, additional questions on the use of Heartweb were included. For example, patients were asked whether they remembered receiving the registration code for Heartweb and how they felt about the procedures and contents.

### Analyses

Baseline differences between groups were tested with 2-sample t-tests, and χ^2^ or Fisher exact tests. Descriptive statistics were used to present data on frequency and duration of site use. We conducted longitudinal data analysis (PROC MIXED) [36] with a compound symmetry covariance structure to assess differences between groups in changes in outcome measurements during the 8-month study period. The power calculations that were based on anthropometry, blood pressure, and cholesterol outcomes are described below. PROC MIXED in SAS (SAS Institute Inc., Cary, NC, USA) takes the dependence of repeated measures of a particular outcome within 1 participant into account. Because of clustering of patients within general practices, the intracluster correlation coefficients (ICCs) of the baseline values of anthropometry, blood pressure, and cholesterol levels were calculated. All ICCs were below 0.001, indicating that the average correlation between outcome variables measured in patients in the same general practices was not different from the average correlation between outcome variables measured in patients in different general practices [37]. The longitudinal analyses were therefore conducted without a random statement for general practice (ie, without taking a possible effect of general practice into account). In addition to the analyses in which all randomized patients were included irrespective of whether or not they had been exposed to the intervention (intention-to-treat analysis), we conducted longitudinal analyses that assessed the differences in effects between users in the intervention and control groups. Because the ICCs were sufficiently low (<0.0002), these analyses too were conducted without a random statement for general practice. All analyses were conducted with the SAS system and *P* values less than .05 were considered statistically significant.

Conservative power calculations (power=.90 and α=.05) based on the study by Van der Veen et al [5] showed enough power to detect differences in change between groups in anthropometry, blood pressure, and serum lipid levels. For example, we were able to detect significant differences in change of as little as 0.35 mmol/L in total cholesterol and approximately 0.5 kg/m^2^ in BMI. However, for differences between the intervention group and the control group to be clinically relevant, (effect sizes that can be obtained with much less time-consuming prescription of medication are generally believed to be larger), they would have to be larger than the aforementioned exemplary critical values of 0.35 mmol/L in total cholesterol or 0.5 kg/m^2^ in BMI.

## Results

### Participant Characteristics

Figure 1 shows the selection and flow of participants. Fifty-five percent of participants were male and participants' mean age was 63 years. Medication use was much less prevalent for type 2 diabetes mellitus (15% of participants) and dyslipidemia (33%) than for hypertension (67%). Most participants were in the maintenance stage of change (Table 1). In comparison with nonresponders (Figure 1), there was a higher percentage of men among the participants. Participants also had statistically and clinically significant higher levels of HDL cholesterol and lower systolic blood pressures (data not shown).

At 4 months, data were available on 66 participants in the intervention group and 68 patients in the control group (Figure 1). Reasons for drop out included illness of participant, death or illness of a family member, refusal to participate further, or movement outside the Kingston area. There was no significant difference (*P*=.18) in drop out between groups and the reasons for drop out seemed unrelated to the (nature of the) intervention. In the intervention group, 3 participants provided no data at 4 months because they were on vacation (n=2) or for personal reasons (n=1). In the control group, 3 participants could not be contacted for measurements after 4 months. In the intervention group, 90% and 84% of participants completed the assessments after 4 months and 8 months, respectively. In the control group, data were obtained for 93% of the participants at both time points. Of the patients who completed the measurements after 8 months, 92% of patients in the intervention group and 80% in the control group returned the evaluation questionnaire.

### Heartweb Use and Users

By and large, Internet use among the participants was highest at home (98% of patients) and at work (27% of participants). Internet use at homes of friends or family, in the library, and at other locations was much lower (9%, 5%, and 2% of participants, respectively). At randomization, none of the patients reported using the Internet to contact other people with hypertension, type 2 diabetes mellitus and/or dyslipidemia. The evaluation questionnaires showed that 93% of participants in the intervention group could recall receiving the result sheet for the baseline measurements, but only 71% could recall receiving the registration code for Heartweb, which was sent with the result sheet. For measurements after 4 months, these rates were 88% and 52%.

Seventeen of the 73 participants (23%) visited Heartweb at least once in the first 4 months of the study. Between 4 and 8 months, 7 additional people visited the site. The 24 Heartweb users visited the site 95 times (range 1-36 times per user, median=1). The median visit duration was 9 minutes 31 seconds and median cumulative visit duration was 16 minutes 56 seconds. Peaks in site use were observed immediately after the result sheets had been sent and in the days immediately before patients were scheduled for their appointments after 4 and 8 months. In total, 33% of patients in the intervention group used Heartweb. Posting of messages to the bulletin board during the study was limited. Most messages on the bulletin board contained requests for factual information directed to the research team. Hardly any patient-patient interaction occurred.

Patients in the intervention group who used Heartweb were significantly younger than those who did not (58 ± 9 vs 64 ± 11 years, *P*=.03). At baseline, there were no differences between users and nonusers with respect to sex (*P*=.32) or in the following anthropometric and biochemical outcomes: BMI (*P*=.35), waist-to-hip ratio (*P*=.24), systolic and diastolic blood pressures (*P*=.77 and *P*=.51), and concentrations of total cholesterol (*P*=.24), HDL cholesterol (*P*=.40), LDL cholesterol (*P*=.33), and triglyceride (*P*=.62).

### Effectiveness of the Intervention

There was no statistically significant difference in change between groups in distribution across stages of change. The prevalence of medication use for type 2 diabetes mellitus, hypertension, and/or dyslipidemia remained stable throughout the study (data not shown). At baseline, there were no differences between groups with respect to social support, anthropometry, blood pressure, and cholesterol levels (Table 2). We observed no statistically significant differences between groups with respect to change in any outcome measurement from baseline to 4 and 8 months. There were no significant differences in change over all 3 time points between groups. Subgroup analyses that compared users of Heartweb with patients in the control group showed similar results (data not shown).

**Table 1:** Baseline characteristics of 146 Canadian patients at increased cardiovascular risk in general practice*

			**Intervention Group** **(n=73)**	**Control Group** **(n=73)**	***P***
**Demographics**					
	Age, mean (SD)		62 (11)	64 (10)	.13
	Male		52	59	.51
**Education Level**					
	Low (≤high school level)		21	18	.14
	Intermediate		42	30	
	High (>BSc level)		37	52	
**Lifestyle**					
	**Smoking Status**				
		Never smoker	35	39	.60
		Ex-smoker	51	52	
		Current smoker	14	9	
	**Alcohol (>3 glasses/wk)**		56	54	.87
	**Exercise (>3 times/wk)**		63	61	.81
**Medication Use for**					
	Hypertension		67	67	1.00
	Dyslipidemia		35	31	.72
	Type 2 diabetes mellitus		13	18	.47
**Stage of Change**					
		Precontemplation	15	16	.21
	Contemplation		3	5	
	Preparation		1	7	
	Action		13	4	
	Maintenance		68	68	

^*^ Values are percentages unless otherwise specified.

**Table 2:** Baseline measurements and changes after 4 and 8 months in anthropometry, blood pressure, and cholesterol levels in Canadian patients at increased cardiovascular risk in general practice*

		**Baseline**						**Change after 4 mo†**				**Change after 8 mo‡**				
		**I**		**C**				**I**	**C**			**I**	**C**			
		**Mean**	**SD**	**Mean**	**SD**	***P*§**		**Mean**	**Mean**	***P*||**		**Mean**	**Mean**	***P*¶**		***P*#**
Social Support**																
	Perceived support	5.7	1.3	5.7	1.2	.87		0.11	-0.08	.29		-0.17	-0.07	.60		.31
	Social network	3.5	0.5	3.5	0.5	.35		-0.06	0.04	.21		0.01	0.07	.49		.45
**Anthropometry**																
	BMI, kg/m^2^	29.5	5.2	29.2	4.5	.73		0.08	-0.21	.07		-0.02	-0.01	.97		.12
	Waist-to-hip ratio	0.91	0.08	0.92	0.07	.25		-0.02	-0.01	.64		-0.004	-0.01	.32		.35
**Blood Pressure, mm Hg**																
	Systolic blood pressure	134	14	136	18	.42		-0.4	-2.1	.46		-1.9	-5.2	.16		.37
	Diastolic blood pressure	81	9	80	11	.61		-0.2	-1.4	.44		-2.5	-3.2	.60		.72
**Cholesterol, mmol/L**																
	Total cholesterol	5.5	0.9	5.4	1.2	.56		0.03	-0.06	.41		-0.08	-0.11	.79		.70
	HDL cholesterol	1.56	0.44	1.47	0.39	.21		0.04	0.02	.59		-0.01	0.01	.28		.27
	LDL cholesterol	3.2	0.9	3.1	1.0	.68		0.06	-0.10	.09		-0.07	-0.10	.73		.20
	Triglycerides	1.9	1.9	1.9	0.8	.90		-0.21	-0.04	.16		-0.02	-0.09	.58		.15

^*^ Data are presented separately for the intervention group (n=73) and the control group (n=73). BMI, body mass index; C, control; HDL, high-density lipoprotein; I, intervention; LDL, low-density lipoprotein.

^†^ Change between baseline and 4 months.

^‡^ Change between baseline and 8 months.

^§^ T-test *P* value for difference between the intervention group and the control group at baseline.

^||^ PROC MIXED *P* value for difference between the intervention group and the control group in change between baseline and 4 months.

^¶^ PROC MIXED *P* value for difference between the intervention group and the control group in change between baseline and 8 months.

^#^ PROC MIXED *P* value for difference between the intervention group and the control group in change between baseline, 4 months, and 8 months.

^**^ The range for perceived support is 1 to 7, and the range for social network is 1 to 5. A higher score indicates higher support levels.

## Discussion

### Principal Findings

This randomized, controlled intervention study failed to show favourable results of a Web-based, targeted nutrition counselling intervention with a social support component on anthropometry, blood pressure, and serum cholesterol levels compared with control. Changes after 8 months tended to be more favourable in the control group than in the intervention group. It is unclear what caused these effects. One possible explanation for the lack of effectiveness could be the low uptake of the intervention, with only 33% (24 of 73) of participants in the intervention group using the Web-based counselling tool. Most participants used the tool only once during a period of 8 months. Nonuse of the intervention by patients in the intervention group has been highlighted by Eysenbach [38] as one of the methodologic challenges of doing a randomized, controlled trial of a Web-directed intervention.

Changes in clinical outcomes were expected to be the effects of dietary changes, which in turn were expected to be the result of changes in motivation and changes in social support. However, no beneficial changes in motivation or social support were observed in the intervention group.

Unfortunately, no reliable data on dietary intake are available in this study. To our knowledge, this is the first randomized controlled trial in which the effectiveness rather than the efficacy of a Web-based nutrition counselling and social support intervention was studied in older patients at increased cardiovascular risk by using clinical outcome measurements. An important strength of the study was the nonselective and low drop out rate; for 91% of participants, data on 2 or more time points were available. This was likely the result of the fact that patients were recruited through their respective general practitioners.

### Comparison with Other Studies

Related studies by Tate et al [39] and Oenema et al [32] in different populations showed improvements in body weight and self-rated fat intake. In those studies, however, high exposure to the intervention was guaranteed by recruiting volunteers through email invitations and by installing the intervention program on a local hard disk. The low exposure to the intervention in our study was likely an important cause for the lack of effectiveness. Further, at baseline, participants had high levels of motivation and support and relatively good health compared with the non responders. Further, use of medication to control type 2 diabetes mellitus, dyslipidemia, and hypertension was frequent. Because current practice guidelines recommend nutrition counselling before the prescription of medication, it is reasonable to assume that most participants already had a history of nutrition counselling. One may wonder how much effect any intervention might have on a highly treated population with relatively good health at baseline. A final explanation for the lack of effectiveness may lie in the generally low effects of nutrition counselling on clinical outcomes [40].

### Study Limitations

We had anticipated social support to be a key factor in the intervention. Nonetheless, despite previous studies showing positive and negative effects of Internet interventions on social support, we found no difference in change in social support during our study period [10,12,41,42]. The absence of changes in social support may have been the result of high baseline support levels. Other possible explanations include the potentially limited effect of extending older people's social networks, a reluctance to ask or provide social support for partly self-inflicted conditions such as being overweight, the relative short duration of the intervention for a long-term stressor such as risk factors for cardiovascular disease, the lack of involvement by a general practitioner, or the use of a random group of peers rather than peers matched by personal characteristics such as age, sex, level of education, religious beliefs, and type of stressor [43-45]. Another intermediate outcome, motivation to change, showed similarly minor effects. A median of 1 site visit may have been insufficient to produce significant improvements. Moreover, the high percentage of people who were already in the action and maintenance stages at baseline may have limited the possibility of finding improvements. It would be interesting to know whether there were any changes in the dietary fat intake of people in the intervention group who were in the maintenance stage at baseline. Their dietary fat intake at baseline was most likely higher than recommended as a result of common misconceptions. It is possible that participants who classified themselves in maintenance at baseline and did not eat a low-fat diet changed to in maintenance and began eating a low-fat diet as a result of the intervention. Unfortunately we have no dietary intake data to study this possibility.

The percentage of people who used the Web-based counselling tool (33% of people who reported to be regular Internet users and volunteered to be in a nutrition counselling study) was low in comparison with findings by Tate et al [39]. Further, the number of times participants used the site was limited (median=1 visit in 8 months). This is particularly disappointing because Tate et al [39] showed that the effect size in a weight-loss trial was related to the number of site visits. For Web-based tools to have considerable public health impact, it is absolutely necessary to increase the number of users and the frequency of use. This is particularly challenging because of the low percentages of people who are motivated to change their behaviour [46]. Cowdery et al [47], for example, predicted a reach of 22% for targeted Web products. Several approaches may have positive effects on reach and site use. Improved access to computers and the Internet, improved computer skills, a stronger indication of the potential benefits, and improved privacy protection of online information have been suggested to increase the reach [48,49]. Adding a longitudinal component to the site (ie, providing patients with feedback on their progress over time), sending (email) reminders to use the site, and increasing the “fun” component of the site (eg, by including a nutrition knowledge game) may have positive effects on the frequency of site use. However, people's interest in the Internet as a medium for health information may be particularly pronounced only for more stigmatizing or directly life-threatening diseases.

### The Future of Web-Based Approaches in Research and Practice

Because risk factors for cardiovascular disease occur frequently in older people [50], the elderly are a large target group for lifestyle interventions. However, because far fewer older than young people use the Internet, it is understandable that older people in particular are somewhat hesitant to use these innovative approaches. In addition to the narrowing digital divide, this attitude will likely change over time, thus increasing the potential of Internet-based interventions [51,52]. As with other technical advancements, it may take decades before the public fully adopts the medium. At present, it is too early to draw definite conclusions on the true use of Internet interventions. Cautious interpretation of our study results is also necessary because use of Heartweb in a real-life situation is different from study participation. Patients who were in principle interested in computer-based approaches may have been discouraged by the burden of the study questionnaires and appointments that are not necessary if Internet-based interventions are applied in real life [53]. The median cumulative site visit duration longer than 16 minutes stresses the potential of Internet-based tools in addition to the short practitioner-patient contact during regular health checks. Approaches to keep patients engaged and involved in interventions over a longer period are therefore necessary.

The lack of controlled trials on the feasibility and effectiveness of the thousands of health education web sites indicates the need for research in this area [54]. It is quite possible that the users of computerized patient education are already the most compliant proportion of patients with practitioner-directed care. However, Internet-based tools can only be exploited to their full extent if they also get through to patients who are otherwise hard to reach and possibly undertreated in regular primary care. For example, people with a low socioeconomic status are usually at higher risk but hard to reach in health-promotion programs. They would benefit from the individualized pace of instruction and the nonthreatening learning that can occur with a computer-based learning program [24,55]. Therefore future research should help identify characteristics of the users of Web-based interventions with respect to compliance and regular care. Similarly, little is known about the factors that determine whether people are likely to be attracted to and benefit from a support group [13,56].

### Implications

Altering patients' dietary behaviour in a sustainable way has repeatedly been shown to be difficult to achieve. As of yet, computer-based interventions have not been the magical breakthrough they were hoped to be. Uptake of the current Web-based intervention was low. Our study showed no favourable effects of a Web-based nutrition counselling and social support tool for patients at increased cardiovascular risk who were likely relatively healthy and more motivated than the general patient population. However, computers can partly take over the burden of continuous care for patients with chronic diseases [57]. Therefore we believe that real-life Internet-based interventions in the future have a true potential, particularly because the continuous contact that is necessary for long-term behaviour change is difficult due to time constraints for patient and clinician. Increasing the uptake of Web-based nutrition counselling interventions remains challenging and is a key factor to successful implementation. To make full use of the possibilities of face-to-face and computer-based interactions, we strongly advocate that the World Wide Web should never fully replace consultations and clinical examinations by general practitioners or other health professionals.
